# Supplementary material for: Modeling glioblastoma heterogeneity as a dynamic network of cell states
Source: Mol Syst Biol. 2021 Sep 16;17(9):e10105. doi: 10.15252/msb.202010105 (PMC8444284; doi:10.15252/msb.202010105)
Supplement: Supplementary file 6 — Source Data for Figure 5 [file MSB-17-e10105-s004.zip › Figure5A_sourcedata/GSEA_3071/nosigdb_stateIII.GseaPreranked.1622795155562/LARSSON_STATE_1.html]

Details for gene set LARSSON\_STATE\_1[GSEA]

|  || Dataset | state33071 |
| Phenotype | NoPhenotypeAvailable |
| Upregulated in class | na\_pos |
| GeneSet | LARSSON\_STATE\_1 |
| Enrichment Score (ES) | 0.38696402 |
| Normalized Enrichment Score (NES) | 3.380408 |
| Nominal p-value | 0.0 |
| FDR q-value | 0.0 |
| FWER p-Value | 0.0 |
Table: GSEA Results Summary

  

Fig 1: Enrichment plot: LARSSON\_STATE\_1      
 Profile of the Running ES Score & Positions of GeneSet Members on the Rank Ordered List

  

| PROBE | GENE SYMBOL | GENE\_TITLE | RANK IN GENE LIST | RANK METRIC SCORE | RUNNING ES | CORE ENRICHMENT || 1 | RPS12 |  |  | 8 | 0.615 | 0.0248 | Yes |
| 2 | SH3BGRL3 |  |  | 26 | 0.505 | 0.0425 | Yes |
| 3 | DSTN |  |  | 34 | 0.490 | 0.0621 | Yes |
| 4 | PFN1 |  |  | 40 | 0.481 | 0.0819 | Yes |
| 5 | RPL8 |  |  | 44 | 0.469 | 0.1016 | Yes |
| 6 | TXN |  |  | 47 | 0.465 | 0.1215 | Yes |
| 7 | S100A11 |  |  | 48 | 0.464 | 0.1418 | Yes |
| 8 | S100A10 |  |  | 102 | 0.409 | 0.1460 | Yes |
| 9 | RPSA |  |  | 108 | 0.405 | 0.1624 | Yes |
| 10 | DRAP1 |  |  | 116 | 0.399 | 0.1780 | Yes |
| 11 | SRM |  |  | 133 | 0.388 | 0.1909 | Yes |
| 12 | TMSB10 |  |  | 148 | 0.376 | 0.2037 | Yes |
| 13 | SMS |  |  | 149 | 0.375 | 0.2201 | Yes |
| 14 | ACTG1 |  |  | 153 | 0.371 | 0.2356 | Yes |
| 15 | S100A6 |  |  | 172 | 0.360 | 0.2467 | Yes |
| 16 | PPP1R14B |  |  | 174 | 0.359 | 0.2621 | Yes |
| 17 | PRELID1 |  |  | 176 | 0.358 | 0.2775 | Yes |
| 18 | SPATS2L |  |  | 184 | 0.351 | 0.2910 | Yes |
| 19 | COTL1 |  |  | 198 | 0.340 | 0.3025 | Yes |
| 20 | C12orf75 |  |  | 203 | 0.337 | 0.3162 | Yes |
| 21 | HINT1 |  |  | 205 | 0.335 | 0.3306 | Yes |
| 22 | LGALS1 |  |  | 230 | 0.323 | 0.3385 | Yes |
| 23 | OAZ1 |  |  | 259 | 0.305 | 0.3447 | Yes |
| 24 | TMSB4X |  |  | 273 | 0.300 | 0.3544 | Yes |
| 25 | TPM4 |  |  | 306 | 0.290 | 0.3589 | Yes |
| 26 | CAST |  |  | 356 | 0.272 | 0.3582 | Yes |
| 27 | MYL6 |  |  | 360 | 0.272 | 0.3693 | Yes |
| 28 | CLIC1 |  |  | 373 | 0.269 | 0.3780 | Yes |
| 29 | GADD45GIP1 |  |  | 384 | 0.265 | 0.3870 | Yes |
| 30 | PHLDA1 |  |  | 589 | -0.259 | 0.3457 | No |
| 31 | CTSC |  |  | 1511 | -0.306 | 0.1219 | No |
| 32 | ANK3 |  |  | 2058 | -0.338 | -0.0040 | No |
| 33 | PDLIM4 |  |  | 2346 | -0.361 | -0.0621 | No |
| 34 | GCHFR |  |  | 2963 | -0.415 | -0.2026 | No |
| 35 | CSRP2 |  |  | 2974 | -0.417 | -0.1870 | No |
| 36 | ARL4C |  |  | 2985 | -0.418 | -0.1713 | No |
| 37 | GAP43 |  |  | 3308 | -0.462 | -0.2340 | No |
| 38 | FBXO32 |  |  | 3482 | -0.501 | -0.2567 | No |
| 39 | DPYSL3 |  |  | 3498 | -0.507 | -0.2384 | No |
| 40 | DCBLD2 |  |  | 3543 | -0.525 | -0.2268 | No |
| 41 | DKK1 |  |  | 3556 | -0.530 | -0.2067 | No |
| 42 | PMEPA1 |  |  | 3737 | -0.657 | -0.2244 | No |
| 43 | TMEFF2 |  |  | 3767 | -0.697 | -0.2014 | No |
| 44 | SCG2 |  |  | 3786 | -0.718 | -0.1746 | No |
| 45 | POSTN |  |  | 3885 | -1.025 | -0.1551 | No |
| 46 | IGFBP5 |  |  | 3895 | -1.082 | -0.1101 | No |
| 47 | IGFBP3 |  |  | 3903 | -1.199 | -0.0595 | No |
| 48 | SPP1 |  |  | 3920 | -1.508 | 0.0023 | No |
Table: GSEA details [plain text format]

  

Fig 2: LARSSON\_STATE\_1: Random ES distribution      
 Gene set null distribution of ES for **LARSSON\_STATE\_1**
